# Impact analysis assessment of UAS collision with a human body

**DOI:** 10.1371/journal.pone.0320073

**Published:** 2025-03-27

**Authors:** Zdeněk Svatý, Pavel Vrtal, Tomáš Mičunek, Tomáš Kohout, Luboš Nouzovský, Michal Frydrýn, Tomáš Blodek, Karel Kocián

**Affiliations:** Department of Forensic Experts in Transportation, CTU in Prague, FTS, Prague, Konviktská, Czechia; King Fahd University of Petroleum & Minerals, SAUDI ARABIA

## Abstract

This study investigates the safety risks of Unmanned Aerial System (UAS) collisions with humans, focusing on impact dynamics through controlled crash testing. Utilizing a custom-designed drop mechanism, 49 impact tests were conducted on a Hybrid III anthropomorphic test device at various velocities and kinetic energies (7–24 m/s and 1–280 J). Tested UASs included a range of designs and weights (20 g to over 1 kg). Results demonstrated limitations in using kinetic energy models and peak head acceleration for injury prediction. The Head Injury Criterion showed greater consistency, reflecting the temporal profile of impacts. Findings revealed that UAS structural fragility reduces energy transfer at higher impacts through deformation and fracturing. This work underscores the need for improved testing protocols and nuanced safety standards.

## 1. Introduction

Dynamic tests or impact analyses (crash tests) currently present the most accurate method for assessing the potential severity of injury or fatality in wide range of applications. The most well-known and extensively utilised crash testing methodologies are performed in the automotive industry. They serve as a means to identify and determine the severity of the potential injuries or to estimate the probability of injury or fatality occurrence. In recent years, the application of crash tests for assessing the potential severity of injury or fatality in UAS collisions with humans has been proposed. Their adaptation for evaluating Unmanned Aerial Systems safety has provided significant insights into the risks posed by drone collisions with humans. However, despite the growing body of work, limitations remain. Many prior studies lack detailed descriptions or reproducible methodologies, and results were never validated by other researchers or testing facilities. The corresponding costs, time demands, equipment, and necessary knowledge present significant limitations that potentially hinder further application.

To address these gaps, this study explores the repeatability and reliability of crash testing methods through a comprehensive series of impact experiments. A drop mechanism was designed to enable precise control over impact scenarios and to facilitate high-fidelity data collection. The aim was to acquire data for the assessment of test repeatability, validation against previously performed tests, and comparison of different types of UAS under similar conditions, such as the effects of similar impact speeds or kinetic energy (KE). The study specifically focuses on validating prior research outcomes, benchmarking them against new experimental datasets, and refining the understanding of collision dynamics.

## 2. Current knowledge and approaches

Crash tests are conducted through controlled acceleration and collision of the selected UAS with various collision partners. Most commonly used collision partners are represented by Anthropomorphic Test Devices (ATDs) (usually a dummy modified for Federal Aviation mandated or Automotive crashworthiness safety testing), simplified ATDs (which consist of selected parts of the full-scale ATD), or Post Mortem Human Surrogates (PMHS). The aim of the ATD is to simulate the human response to impacts, accelerations, deflections, forces, and moments of inertia generated during the collision. It is designed to model the form, weight, and articulation of a human body. The most commonly used ATD for UAS tests is a 50th percentile Hybrid III. Due to the fact that the ATD was designed and validated for automotive testing, its utilisation for UAS crash testing introduces limitations and a need for a conservative assessment of the observed response. It has been shown [1] that the Hybrid III and the human body have different neck responses because of differences in neck biofidelity. The Hybrid III neck is less compliant than the human neck in the vertical direction, resulting in a greater transfer of force and moment to the neck system. A comparison of inverted drops [2–4] and human cadavers showed that the dummy neck is two to four times stiffer than human cadavers. Furthermore, the Hybrid III crash dummy transmits about 70–75% of the applied force from the head or upper neck to the lower neck area. In contrast, only about 20–30% of the applied force is transmitted from the head to the lower neck on a human cadaver. Still, the ATD currently presents the most accurate tool that can be used to evaluate and assess potential consequences of a collision with the UAS. Performed tests from other studies show [5] that ATD can distinguish the stiffness, structure, and energy transmission of the impacting object.

An impact with a UAS, or its parts, can cause a wide range of injuries, both mechanical (such as trauma, lacerations, and penetration) and non-mechanical (such as burns and overpressure). In this study, the focus is only on blunt traumatic injuries, similar to previous research [5–10]. A significant aspect of quantifying and identifying the risks arising from a UAS collision with a person, based on the collision partner’s response, is the criteria describing human vulnerability. Most of the currently used HVMs (Human Vulnerability Models) are based on or derived from military research, mainly the protection of individuals from projectiles and fragmentation caused by explosions [11,12], falling debris [13], or non-lethal ammunition [14], or utilize criteria and prediction models from the automotive industry to assess the resulting rate of injury severity or its probability [5–9]. The standard method for evaluating the severity of injuries is the Abbreviated Injury Scale (AIS), a widely used anatomical coding system created by the Association for the Advancement of Automotive Medicine [15]. The system is commonly used to classify the severity of injuries and represents the threat to life (AIS 1 being a minor injury, AIS 3 being a serious injury, and AIS 6 being a fatal injury).

Out of the identified studies, only a limited amount of the presented tests were supplemented with sufficient details or resulting data. The main outcomes and conclusions are listed to enable a deeper understanding of the issue and its limitations. The authors conducted a set of tests prior to this study [9], involving five small UASs, including three multi-rotor quadcopters and two fixed-wing aircrafts. The purpose of these tests was to verify the current methods and serve as a basis for assessment. Results obtained through the crash tests indicate that the currently used kinetic energy threshold values are excessive and that some proposed criteria, such as the blunt criterion, have limited value. On the other hand, the results suggest that the car vulnerability criteria are appropriate for use.

Another example is Koh et al. [16], who used practical tests for the validation of Finite Element Method (FEM) models. A simplified ATD device was used (50th percentile male Hybrid III head with springs attached to a solid surface) for evaluating the severity of an impact. Only the mass (from 305 up to 5100 g), drop height (10, 20, 30, 50, and 100 ft) and head acceleration were recorded. Two types of custom UASs were used. Smaller PA66 plastic frames and larger carbon fibre frames were adjusted to enable the mounting of additional weights to achieve the required weight. The UASs were dropped with the use of electromagnet and impacted the simplified ATD with its underside. For sUASs under 2 kg (the scope of this study), the resulting Peak Head Acceleration and Head Injury Criterion (HIC15) correlated with increasing KE, with increasing values corresponding to greater weight and height. The smaller PA66 frame impacts led to higher values of the head acceleration and HIC15 (820 g; 15 m; Peak Head Acceleration 388 g, HIC15 1023) than the larger carbon frame (1400 g; 30 m; Peak Head Acceleration 233 g, HIC15 272). The corresponding probabilities of a skull fracture (AIS 2) were 48% and 5%, based on the FMVSS.

Campolettano et al. [17] performed live flight and falling impact tests using an instrumented Hybrid III test dummy (which measured head and upper neck). DJI Phantom 3, DJI Inspire 1 and DJI S1000 + were tested in two kinds of tests: flight tests and falling impact tests. In the flight tests, an operational UAS was flown (at speed achieved after 40 m distance) into the head of the Hybrid III test dummy while a non-operational UAS was dropped from a height of 5.5 m onto the head of the Hybrid III test dummy for the falling impact tests. Batteries were removed from the UAS for these falling impact tests and replaced with an equivalent mass. Between 1 and 3 flight impact tests were conducted for each UAS and between 5 and 7 for the falling impact tests. The necessary accuracy of the flight tests was not achieved, and thus only a limited number of flight tests achieved the desired impact configuration. Impact orientations varied between the falling impact tests. The achieved velocity and mass of the tested UASs were not provided. Furthermore, the individual test results were aggregated into minimum, maximum and average values for Peak linear acceleration, HIC15 and Nij. During the falling impact tests, a variety of UAS impact orientations were investigated, including direct impacts from the base of the UAS as well as indirect impacts from the arms or legs of the UAS.

At NIAR at the Wichita State University [5], 26 tests were performed with a DJI Phantom 3 Standard (2.7 lb) impacting the 50th percentile male Hybrid III. The UAS was accelerated by a drop sled or pendulum and collided with the top of the head of the ATD (13x horizontal positions – failure at hover, 3x vertical, 4x under an angle of 65° and 6x under 58°– failure at forward speed). The test impact speeds varied from 17 to 50 ft/s (5 – 15 m/s), and the resulting KE varied from 12 to 106 ft-lb (16 – 144 J). Automotive criteria were used for the evaluation of the severity of the injury. The highest estimated probability of an AIS 3 neck injury (Nij 12.5%) was observed at a vertical impact with an impact speed of 43.2 ft/s and a KE of 77.4 ft-lb (105 J). Overall, the tests showed a very low probability for a skull fracture based on HIC15. The highest measured Peak Head Acceleration was 139 g during impact under 58°. Due to the high discrepancy between the estimated probability of injury from the automotive criteria and the predicted PoF by the RCC 321 risk curves (99% PoF for more than 12 tests), tests were also performed with wood (3 tests) and steel blocks (3 tests). The tests were performed with a similar impact configuration and impact KE (approximately 120 ft-lb; 160 J) as those used in the tests with the highest impact velocity. The impacts resulted in high Peak Head Acceleration values (1000 g) and HIC15 (above 8000). These values are outside the ranges of the datasets used for the determination of the automotive probability models. Thus, despite the inability to predict a resulting injury, the high values correlate well with the prediction of a high severity from the RCC 321 risk curves. Furthermore, these tests also showed that the ATDs are able to differentiate the impacting object’s rigidity, structure and potential energy transfer.

In the A14 Report [6–8], the tests were performed with different UASs (multi-rotor, fixed wing, influence of a parachute), under various impact scenarios and with different impact partners – a simplified ATD [5] (Head and neck of 50th male Hybrid III fixed to a solid surface), an ATD [7] (50th male FAA Hybrid III) and a PMHS [8]. However, the detailed data and tests results were available only for 149 tests, which are used for the purpose of this study. The simplified ATD was used to evaluate the worst-case impact orientations at 25 and 36 ft/s (7.6 and 10.9 m/s). The tests with ATD were near terminal velocity to estimate the severity of the impacts and the PMHS tests were used for the verification of the obtained results. A drop sled, pneumatically actuated cylinder and elastic band were used to accelerate the UAS. The simplified ATD test setup showed limits to a maximum impact speed, a higher resulting Peak Head Acceleration (1.35 – 1.48x), a different response with the use of rigid impactors (such as a wood block) and a different response to the impacting of a foam fixed wing UAS. While it can be used to determinate the worst-case impact orientation for multi-rotor UASs, due to the higher rigidity, it does not appear suitable for the evaluation of the potential resulting injury severity. For the ATD and PMHS, the impacting configuration varied between a horizontal, vertical and angled (58 and 80°) direction of the UAS to the top, front, back and side of the head. Various impact locations on the UAS were tested (mostly the identified worst-case orientations for the given UAS). However, horizontal impacts to the head for the multi-rotor UAS seem contradictory to previous work and conclusions, which assumed that impacts for a multi-rotor UAS occurred only at steep angles above 55°. While the results of the direct horizontal impacts to the head illustrate that the UAS presents a risk under a high velocity, such an impact configuration seems improbable. A difference between the measured acceleration was observed with redundant accelerometers during the PMHS tests (up to 31%), likely due to the sensors’ mounting position and the resulting deformation of the skull during impact. Furthermore, the measured values for the PMHS tests were higher than for the same ATD tests.

An alternative approach is the utilisation of numerical modeling. Computer-based modeling can be used to examine energy transfer, human body response, forces on the tissue, and energy absorption by the UAS structure in great detail, with the ability to easily change various parameters. The model can be represented either as a multi-body system or through the Finite Element Method. The computation simulation represents the most suitable solution for rapid validation and safety assessment. The option to easily change the tested scenarios, observe the effects of probable impact configuration or easily repeat and validate various measurements. There are several studies which focused on numerical modeling of the UAS impact [11]. However, the accuracy and validity of such simulations are inherently dependent on the used models, available information about the UAS and its characteristics and most crucially on their validation against performed crash tests. This fact represents one of the main limitations and restricts their interpretive value today.

### 2.1. Test procedures and standards

Before starting the testing, it was necessary to know the different approaches found in the current standards related to UAS testing. The following section introduces the basic standards from the EU, USA and Canada.

In the EU, only relevant standard is the prEN 4709 which does not provide or specify details for crash testing of the UAS to assess safety in case of a collision with an UAS. While crashworthiness is considered within the standard, the focus is on mitigating the risks of explosions, fire hazards and the cutting hazards from the structure or propellers in case of impact. The test procedures are performed only at the UAS’s maximum horizontal velocity.

In the USA, the ASTM F3389/F3389M-21 [18] provides four methods for evaluating the potential for impact injury: a simple analytical method (Method A); a simplified test (Method B); a more rigorous test (Method C); and a test method normalized to approximate energy transfer values (Method D). Only Methods B and C are utilizing pure UAS crash testing for the assessment and will therefore be described further. The standard does not specify exact threshold values and allows the governing CAA to define them based on specific applications.

Method B uses an instrumented simplified ATD (head and neck of a 50th percentile male Hybrid III) to determine a relation between acceleration and the impact KE of the UAS. The applicant must conduct a series of impact tests for the identified MPWC scenario. At least ten drop tests should be performed, with five for each of the two defined drop heights. The heights vary based on the weight of the UAS and the use of parachute mitigations. For sUASs with a mass below 2.2 lbf (1 kg), the drop heights are defined as 10 and 20 ft (approximately 3 and 6m). For heavier UASs (up to 8 lbf), the impact KE should be 20 and 40 ft-lbf (approximately 27 and 54 J). For parachute testing, the impact velocity should be 8 and 16 ft/s (2.5/5 m/s). Subsequently, a linear fit with a forced zero intercept should be defined and recorded as an energy transfer slope (S). The maximum safe KE is then determined by limiting the maximum accepted peak head acceleration.

Method C uses full-scale ATD (50th percentile male Hybrid III) to determine the energy transfer slope (S), HIC15, Nij, and upper neck compression values. The crash tests should involve at least two tests for three of the following impact directions: a vertical drop, a horizontal impact to the head, and an angled impact to the front of the head. This requires the use of a combination of testing facilities, enabling not only the drop test but also angled and horizontal impacts. All tests should be performed at the MPWC orientation, and the impact speed varies depending on the type of test. Vertical tests should be performed at the identified critical speed defined by ASTM F3341/F3341M – 22, which is the same as in AC 922-001 (maximum cruise speed for fixed-wing UAS and terminal velocity for multi-rotor UAS). The horizontal test should be performed at the operational speed of the UAS. If the testing facilities cannot achieve the required impact speeds, the testing must be performed at least three times at evenly distributed impact speeds. Therefore, a total of twenty-seven tests must be performed instead of six.

Canadian AC 922-001 is supplemented by Appendix C, which provides guidance on the methodology for assessing UAS safety. The Circular describes six basic types of dynamic test procedures: one test where the predominant impact vector is vertical, three tests where the dominant impact vector is horizontal, and two tests using a worst-case vector defined by flight testing showing different failure conditions. The same tests are defined for various types of UAS (multi-rotor, fixed-wing, rotary-wing, hybrid, or lighter-than-air). At least two tests should be performed for each of the following test configurations: Vertical Drop Test (at critical speed and normal flight orientation), Frontal Head Test (at operational speed and normal flight orientation), Head Critical Impact Direction (at critical orientation and speed), Head Side Impact (at operational speed and normal flight orientation), and Chest Critical Impact (at critical orientation and speed).

In contrast to the ASTM, Appendix C also specifies that the ATD be seated in a rigid position to obtain conservative results and control variability. Additionally, the seat should be rigid to avoid any deformation that may alter the test results. Test facilities such as a launcher, sled tester, or drop tower may be used and enable the possibility to adjust the position of the ATD based on the chosen test configuration and facility used. Automotive criteria are used to assess the test results, along with the defined threshold values. Suggested threshold values derived from automotive crashworthiness tests are also defined for chest injury. Notably, the set limits for peak head acceleration (237 g) and Nij (1.21) correspond to a 30% chance of injury and differ from standard aviation and automotive limits (200 g and 1).

### 2.2. Peak head acceleration

**Fig 1:**
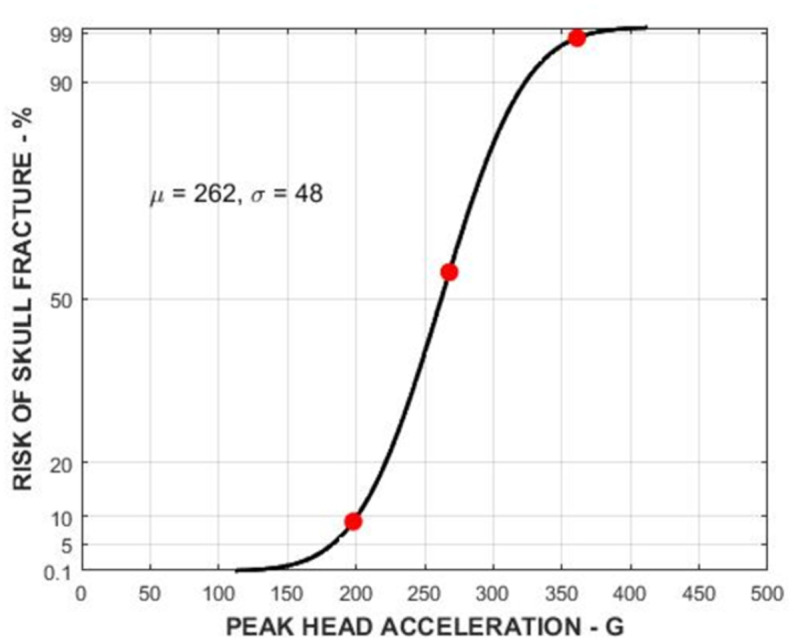
Skull fracture risk curve for the adult population based on the Peak Head Acceleration with the 198 g, 268 g and 361 g.

Apart from the commonly used criteria in the automotive industry, such as the Head Injury Criterion (HIC), peak head acceleration over 3 ms or the Neck Injury Criterion (Nij), the UAS standards consider the utilisation of maximum peak head acceleration as a descriptive tool for estimating the consequences of impact (through the energy transfer slope). This is primarily based on research conducted by the Assure Group [4,6]. Initially, it was proposed as a 3σ trend line (99.7%) to express the relationship between KE and Peak Head Acceleration to evaluate the potential severity of head impacts (Fig 1). This relationship, specific to the given UAS, was further explored in [5,6]. The most recent changes to the approach were incorporated into ASTM F3389/F3389M-21 [18] in Methods B and C for assessing the safety of a sUAS.

Initially, a peak threshold value of 198 g was also established for a skull fracture, based on research by Yoganandan [17] using a 48mm radius hemispherical anvil impactor to study skull fractures (6 quasi-static and 6 dynamic tests on human heads from various angles). However, based on Mertz [19–21], this value represents only a 9% probability of a skull fracture for the adult population. This was confirmed in the A14 Report [6], which concluded that, based on experimental data (ATD and PMHS), the 198g threshold is considered overly conservative. This was subsequently adjusted in the final A14 report to a value of 237g which corresponds to a 30% chance of skull fracture (AIS 2). Yet, if data from all Yoganandan tests [17] are considered (6 tests, average force necessary for a skull fracture, and average PMHS head weight), the resulting head acceleration value for fracture is 361 g. This value correlates with Mertz research [19], with an approximate 98% chance of a skull fracture, as shown in Fig 1. If this value is adjusted to the average measured force and the Hybrid III ATD head mass, the resulting acceleration is 268 g (approx. 55%).

**Fig 2:**
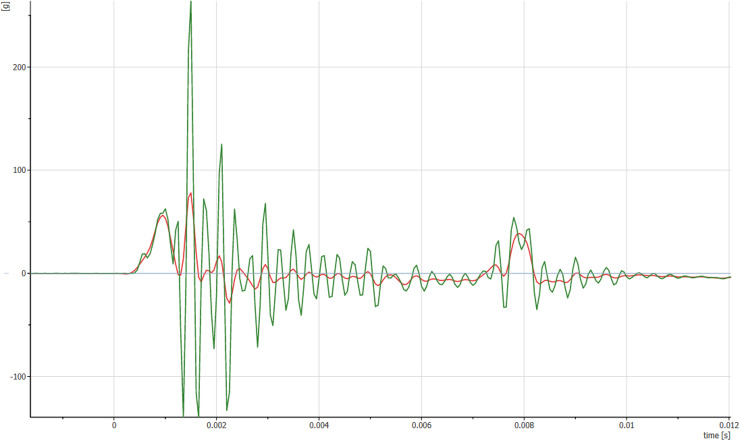
Un-filtered and filtered signal from the accelerometer placed in the CG of the ATD (tests sample from CTU used ATD during an impact test with sUAS).

As mentioned earlier, the maximum observed peak head acceleration is not commonly used in the automotive industry. While the ANM-03-115-31 utilizes the maximum value of 200 g for blunt trauma assessment, this value is not explained further. The authors of this study believe that there is a potential issue with the use of the maximum peak head acceleration. While it can be used to predict a skull fracture, the effects of acceleration and related severity are always interconnected with the time duration over which they affect the subject. Therefore, time is considered in the 3 ms limit or HIC assessment. Furthermore, the proposed assessment in Method B utilizes a simplified ATD, which has an inherently stiffer response. The comparison tests performed in the A14 Report showed a higher observed peak head acceleration (up to 1.5x). In combination with the utilization of threshold values determined for full-scale ATD or adult populations, this may lead to overly restrictive predictions. Furthermore, the maximum peak head acceleration is observed as a response from the ATD. The potential sampling frequency or resonance can significantly affect the resulting values. To eliminate potential bias and other factors, the data must be filtered prior to assessment. The most common filter used in the automotive industry is the CFC1000. To date, no research has been performed to validate its use for UAS impacts, so even its application may not be the most suitable for analysis. The effects of the above-mentioned parameters on the data can be shown in the Fig 2.

**Fig 3:**
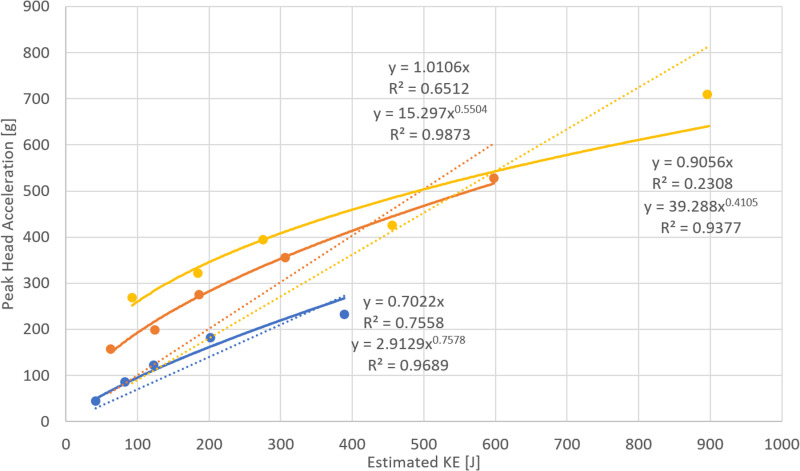
Relation between the estimated KE at the impact and resulting Peak head acceleration for test performed by Koh et al. (carbon frame UAS with mass of 1,4 (blue), 2,1 kg (orange) and 3,1 kg (yellow) with linear (dotted lined) and power trend fitting (solid line).

Furthermore, the available data from Koh et al. [2] show that the relation between the KE and Peak Head Acceleration may not be linear for certain impact configurations. The tests performed within their research used two types of custom UASs (carbon and PA66 frame). The UASs’ mass was gradually increased, and for each series of drop tests, the tests were performed on the top of a simplified ATD (Hybrid III head mounted via springs). Peak head acceleration and HIC15 were measured and subsequently used for validation of the performed computational simulations. While the achieved impact speed was not stated, the drop height and mass were provided for each test. Based on the data used for subsequent simulation (the effective area, drag coefficient, and air density), the impact speed and resulting KE could be estimated. Only three out of the tested configurations with a carbon frame had sufficient number of tests to enable the estimation of the relation between the KE and Peak Head Acceleration. These tests are shown in Fig 3, which presents the achieved impact KE and measured peak head acceleration. For these tests, two types of trends were determined: a linear fit with forced zero intercept (as defined in the ASTM F3389/F3389M-21) and a power fit. Fig 3 shows the results together with the estimated equations of trend fitting, their formulas and resulting R^2^ values.

Although there is only a limited amount of data and more tests are necessary for a conclusive decision, the results indicate that the power relation may be more suitable for some UAS configurations. A linear trend determined with forced zero intercept through three tests is more conservative and less demanding, as only three tests can be used to define the slope (as defined in the standard). However, the question is whether the manufacturer or applicant, when using crash testing to validate the UAS safety, should be required to use a less accurate and more conservative approach. The relation between the peak head acceleration and KE was, therefore, further assessed within this study.

## 3. Method

The aim was to verify the repeatability of the tests, validate the obtained data and further broaden the amount of available information related to drone impact testing. Similarly, to the authors’ previous study on non-destructive testing [9], a certain level of simplification and limitations had to be introduced due to high demands and complexity of the testing.

A vertical fall due to a power system failure for multi-rotor sUASs was selected as the impact scenario. While this does not directly imply a most probable worst-case scenario, the acquired information still enables us to fulfil the defined aims of the testing and to utilise the data obtained from the non-destructive tests. Therefore, the tests were limited to multi-copter sUAS and the testing procedure war restricted to drop testing. Horizontal or angled tests were not performed. While this presents a significant limitation, it allowed us to compare the test results not only with the findings from the A14 Report [6], but also with tests form the A4 Report [5].

In total, 49 tests were performed with various sUASs. The test articles were selected to represent not only the UASs for which test data are already available (from other researches or performed non-destructive tests), but also to cover the identified trends in sUAS development, similarly to the non-destructive tests [11]. The tests were performed at the Center for Experimental Geotechnology – The Josef Complex, Czech Republic.

The set of UAS tested included a total of 19 specific types, ranging from a weight of about 20 g to drones at the boundary of the so-called “harmless” category corresponding to 250 g, and larger machines with weights exceeding 1 kg. The tested drones consisted mainly of machines from the DJI brand, which are the most frequently represented in the tests already conducted, thus allowing for the validation of the chosen testing methodology and the outputs obtained. Due to the gradual development of supply on the market, even more compact types (e.g., DJI Spark, DJI Mini) were included, reflecting the trend of decreasing mass and increasing compactness and stiffness of the central components with folding arms. A non-negligible part of the UAS tested included drones with different design or materials (such as FPV carbon frames). The range was extended not only to include machines from other manufacturers, but effort were made to incorporate clones widely available on the internet. Each UAS was weighed and photographed prior to the test. Photographic documentation was also performed after the impact to capture the effects of the impact on the UAS and ATD.

An anthropomorphic testing device (ATD) was used for testing. While ASSURE used a modified FAA Hybrid III for Federal Aviation mandated crashworthiness safety testing, the standard 50th male Hybrid III used in automotive crashworthiness was employed in this study.

The ATD was fitted with the high speed Kistler instrumentation, where 27 channels were measured. These include head, chest, and pelvis accelerometers with a range of 1 500 g; measurement of force and torque in the upper and lower parts of the neck; chest compression; lumbar force; and knee deflection. The weight of the dummy is 77.7 kg, with a sitting height of 884 mm. During the tests, the ATD was placed on a specially created chair made of stiff and rigid materials, which prevented any negative effects on the performed measurements. The chair allows the positioning of the ATD between 0° and 90°. For assessing the contact area during the impact, the head of the ATD was covered with a swim cap, which showed depressions shortly after the impact that could be marked and used for subsequent analysis. The effect of the cap was tested prior to the actual tests and was estimated to be minimal. The posture of the ATD was set with the head CG in the middle of the impact zone and directly above the lower neck.

**Fig 4:**
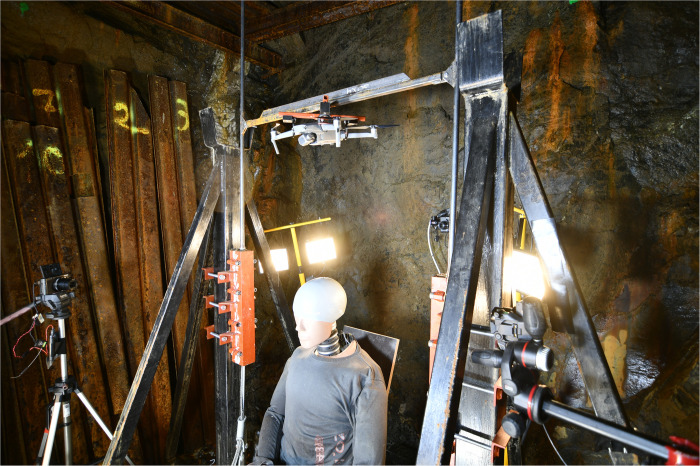
Test setup and drop mechanism.

To achieve the required velocity and collision configuration, a drop mechanism was developed to ensure guidance and stabilization of the tested UAS together with its subsequent release prior to the test (Fig 4). Thus, the UAS is gradually accelerated due to gravity and then released above the head of the ATD. The UAS can be mounted onto the drop sled in either a horizontal position (flight position, head down) or a vertical position. Additionally, a remotely controlled release mechanism was designed to allow the UAS to be dropped from a predefined height (from 30 cm up to 41 m) to ensure the required impact speed or KE. Furthermore, a breaking system was developed to eliminate any effects of the drop sled on the actual test.

**Fig 5:**
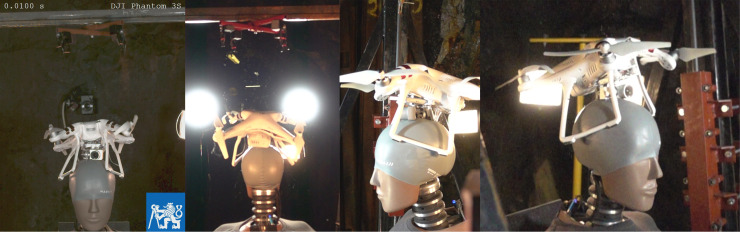
Tests impact configuration.

The tests were captured with five high-speed cameras. The first camera captured the front side of the impact with a frame rate of 2000 fr/s and was backed by a second camera with a frame rate of 500 fr/s. Camera three captured the right side (frame rate of 1000 fr/s), camera four the backside view (1000 fr/s) and fifth camera captured a 45° view between camera one and three (1000 fr/s). The impact velocity was subsequently determined from the recordings of cameras one and three, along with a laser time-gate. An example of a crash test can be seen in Fig 5.

Drop tests were performed at various impact speeds (7–24 m/s) and energies (1–280 J), with drop heights varying from 5 to 37 m. The various test settings and impact configurations were based on the objectives for which the dataset was obtained. These aims were to validate the testing facility against available measurements, assess the repeatability of the test with the same settings and test articles, and evaluate the potential compatibility with other available data. Some of the tests were carried out for estimated terminal velocities of specific UASs. Other tests examined the consequences of various UAS types or structure materials (carbon vs. plastic). Lastly, the effects of ATD orientation were also considered. The final impact configurations were based on available data, conclusions from the non-destructive testing, and potential issues identified in previous parts of this study.

## 4. Results

Tables 1–3 summarize the overall tests results. Test 48 with Mavic 2 Pro was omitted from further processing due to the absence of the desired impact configuration, which only resulted in slight grazing of the front side of the ATD head.

**Table 1:** Crash tests results – Tests impact configuration.

Internal ID	UAS model	UAS mass	Impact Trajectory to Head	Head Impact Location	Vehicle Orientation to Head	Height	Impact velocity	Impact KE
**Unit**	**-**	**kg**	**-**	**-**	**-**	**m**	**m/s**	**J**
**Limit value**	**-**	**-**	**-**	**-**	**-**	**-**	**-**	**80**
**CTU_21_01**	**Tiny Whoop**	0.03	Vertical	Top	Bottom Into Head	5.00	8.78	1.08
**CTU_21_02**	**Tiny Whoop**	0.03	Vertical	Top	Bottom Into Head	10.00	12.11	2.05
**CTU_21_03**	**Tiny Whoop**	0.03	Vertical	Top	Side Into Head	15.00	15.97	3.57
**CTU_21_04**	**Tiny Whoop**	0.03	Vertical	Top	Bottom Into Head	16.10	13.50	2.55
**CTU_21_05**	**DJI Tello**	0.09	Vertical	Top	Bottom Into Head	10.00	12.95	7.21
**CTU_21_06**	**DJI Tello**	0.09	Vertical	Top	Bottom Into Head	16.10	16.33	11.47
**CTU_21_07**	**DJI Tello**	0.09	Vertical	Top	Side Into Head	21.00	20.08	17.33
**CTU_21_08**	**DJI Tello**	0.09	Vertical	Top	Bottom Into Head	20.00	17.81	13.65
**CTU_21_09**	**DJI Tello**	0.09	Vertical	Top	Bottom Into Head	21.00	18.47	14.66
**CTU_21_10**	**Eachine E58**	0.09	Vertical	Top	Bottom Into Head	10.00	11.67	6.06
**CTU_21_11**	**Eachine E59**	0.09	Vertical	Top	Bottom Into Head	18.00	15.77	11.06
**CTU_21_12**	**Eachine E60**	0.09	Vertical	Top	Bottom Into Head	25.00	19.36	16.68
**CTU_21_13**	**Syma X5C**	0.10	Vertical	Top	Bottom Into Head	5.00	7.76	2.98
**CTU_21_14**	**Syma X5C**	0.10	Vertical	Top	Bottom Into Head	12.00	12.63	7.89
**CTU_21_15**	**Syma X5C**	0.10	Vertical	Top	Bottom Into Head	25.00	18.25	16.48
**CTU_21_16**	**Syma X5C**	0.10	Vertical	Top	Bottom Into Head	25.00	18.35	16.67
**CTU_21_17**	**Syma X5C**	0.10	Vertical	Top	Bottom Into Head	25.00	18.48	16.90
**CTU_21_18**	**Eachine E520s**	0.24	Vertical	Top	Bottom Into Head	10.00	13.03	20.21
**CTU_21_19**	**Eachine E520s**	0.24	Vertical	Top	Bottom Into Head	25.00	19.81	46.89
**CTU_21_20**	**Eachine E520s**	0.24	Vertical	Top	Bottom Into Head	37.00	23.49	65.66
**CTU_21_21**	**Meffie Nano**	0.13	Vertical	Top	Bottom Into Head	19.00	17.12	19.20
**CTU_21_22**	**Meffie Nano**	0.13	Vertical	Top	Bottom Into Head	37.00	23.89	37.38
**CTU_21_23**	**RR Calypso**	0.30	Vertical	Top	Bottom Into Head	25.00	20.04	59.83
**CTU_21_24**	**Cinewhoop**	0.49	Vertical	Top	Bottom Into Head	14.50	15.66	59.46
**CTU_21_25**	**Cinewhoop**	0.49	Vertical	Top	Bottom Into Head	25.00	20.13	98.22
**CTU_21_26**	**FOYU FO-F708**	0.15	Vertical	Top	Bottom Into Head	12.00	14.02	14.65
**CTU_21_27**	**RR Calypso**	0.30	Vertical	Top	Bottom Into Head	21.00	18.97	53.63
**CTU_21_28**	**DW686**	0.12	Vertical	Top	Bottom Into Head	12.00	13.72	11.30
**CTU_21_29**	**DJI Spark**	0.31	Vertical	Top	Bottom Into Head	20.00	18.00	49.41
**CTU_21_30**	**DJI Air**	0.44	Vertical	Top	Bottom Into Head	20.00	18.32	72.99
**CTU_21_31**	**DJI Mavic Pro 2**	0.92	Vertical	Top	Bottom Into Head	20.00	17.99	148.32
**CTU_21_32**	**DJI Mavic Pro 2**	0.92	Vertical	Top	Bottom Into Head	25.00	20.08	185.13
**CTU_21_33**	**DJI Mavic Pro 2**	0.91	Vertical	Top	Top	25.00	19.88	180.52
**CTU_21_34**	**DJI Mavic Pro**	0.75	Vertical	Top	Top	20.00	18.22	124.52
**CTU_21_35**	**DJI Mavic Pro**	0.74	Vertical	Top	Bottom Into Head	20.00	18.14	122.40
**CTU_21_36**	**DJI Mavic Pro 2**	0.92	Vertical	Top	Bottom Into Head	20.00	18.13	151.12
**CTU_21_37**	**DJI Mavic Pro 2**	0.91	Vertical	Top	Bottom Into Head	20.00	18.08	149.28
**CTU_21_38**	**DJI Phantom 1**	1.27	Vertical	Top	Bottom Into Head	13.50	15.10	144.16
**CTU_21_39**	**DJI Phantom 3S**	1.22	Vertical	Top	Bottom Into Head	13.00	15.07	138.98
**CTU_21_40**	**DJI Phantom 3S**	1.22	Vertical	Top	Bottom Into Head	20.00	17.96	196.08
**CTU_21_41**	**DJI Phantom 2V+**	1.27	Vertical	Top	Bottom Into Head	20.00	18.21	209.70
**CTU_21_42**	**DJI Phantom 2V+**	1.27	Vertical	Top	Bottom Into Head	25.00	19.92	250.90
**CTU_21_43**	**DJI Phantom 4 Pro**	1.38	Vertical	Top	Bottom Into Head	13.50	14.83	151.82
**CTU_21_44**	**DJI Phantom 4 Pro**	1.39	Vertical	Top	Bottom Into Head	20.00	17.65	215.95
**CTU_21_45**	**DJI Phantom 4 Pro**	1.38	Vertical	Top	Bottom Into Head	25.00	20.10	279.09
**CTU_21_46**	**DJI Phantom 4 Pro**	1.38	Vertical	Top	Bottom Into Head	20.00	18.00	223.88
**CTU_21_47**	**DJI Phantom 4 Pro**	1.38	front 58	58.00	between arms front	20.00	19.04	250.36
**CTU_21_48**	**DJI Mavic Pro 2**	0.92	front 58	58.00	between arms front	20.00	18.08	150.42
**CTU_21_49**	**DJI Mavic Pro 2**	0.92	front 58	Top	Bottom Into Head	20.00	18.06	149.96

**Table 2:** Crash tests results – Measured values.

Internal ID	UAS model	Maximum Resultant Head Acceleration	Head Accel. (3ms)	Head injury criterion (HIC15)	Neck injury criterion (Nij)	Upper neck tension (Fz)	Upper neck compression (-Fz)	Upper neck flexion (-My)	Upper neck extension (My)	Upper neck shear
**Unit**	**-**	**(g)**	**(g)**	**- **	**-**	**N**	**N**	**Nm**	**Nm**	**N**
**Limit value**	**- **	**200**	**80**	**700**	**1**	**6806**	**6160**	**310**	**135**	**3100**
**CTU_21_01**	**Tiny Whoop**	7.27	0.35	0.03	0.02	14.61	81.65	0.81	1.14	31.34
**CTU_21_02**	**Tiny Whoop**	8.86	1.25	0.09	0.03	44.61	167.90	0.97	0.35	31.21
**CTU_21_03**	**Tiny Whoop**	3.31	0.68	0.01	0.02	28.04	107.39	0.41	0.29	15.48
**CTU_21_04**	**Tiny Whoop**	5.62	0.46	0.02	0.00	31.79	124.03	0.69	0.49	17.10
**CTU_21_05**	**DJI Tello**	14.53	3.07	0.63	0.11	72.77	473.58	3.42	5.66	99.13
**CTU_21_06**	**DJI Tello**	58.69	6.94	6.71	0.13	80.02	642.85	3.96	4.56	161.75
**CTU_21_07**	**DJI Tello**	19.20	3.88	0.85	0.02	99.01	507.57	2.90	2.71	89.73
**CTU_21_08**	**DJI Tello**	84.19	3.33	13.58	0.16	116.34	762.99	4.12	6.87	209.28
**CTU_21_09**	**DJI Tello**	74.04	2.53	11.90	0.14	100.17	749.83	3.94	5.16	199.05
**CTU_21_10**	**Eachine E58**	18.92	3.51	0.51	0.06	11.51	314.23	2.52	0.82	74.49
**CTU_21_11**	**Eachine E59**	36.75	2.09	1.87	0.07	62.49	441.35	3.25	1.80	120.58
**CTU_21_12**	**Eachine E60**	57.30	6.78	5.71	0.11	92.16	604.76	4.16	1.31	160.87
**CTU_21_13**	**Syma X5C**	5.74	1.34	0.07	0.06	55.68	316.72	2.48	2.58	40.79
**CTU_21_14**	**Syma X5C**	11.01	2.57	0.31	0.07	66.50	445.64	3.06	2.31	62.85
**CTU_21_15**	**Syma X5C**	20.60	4.62	1.29	0.11	85.53	654.34	3.52	3.93	96.47
**CTU_21_16**	**Syma X5C**	17.95	4.51	1.35	0.10	49.19	592.32	3.79	4.54	96.96
**CTU_21_17**	**Syma X5C**	23.69	5.67	1.17	0.11	88.00	621.86	3.68	4.47	89.56
**CTU_21_18**	**Eachine E520s**	28.47	8.66	4.03	0.14	100.61	811.04	3.99	1.36	137.66
**CTU_21_19**	**Eachine E520s**	55.97	12.77	11.86	0.21	27.13	1211.49	6.65	2.48	220.34
**CTU_21_20**	**Eachine E520s**	67.46	13.09	17.38	0.24	76.09	1422.27	7.31	1.55	261.80
**CTU_21_21**	**Meffie Nano**	68.20	6.29	11.30	0.12	101.97	753.72	4.06	3.30	190.22
**CTU_21_22**	**Meffie Nano**	63.62	11.89	17.17	0.18	125.98	1082.02	5.53	3.56	200.84
**CTU_21_23**	**RR Calypso**	104.25	20.30	46.57	0.36	145.75	2079.45	9.50	9.57	411.39
**CTU_21_24**	**Cinewhoop**	108.42	25.10	62.34	0.44	189.40	2556.21	9.95	3.20	443.38
**CTU_21_25**	**Cinewhoop**	170.05	17.43	169.34	0.52	218.82	3007.08	11.63	5.98	599.04
**CTU_21_26**	**FOYU FO-F708**	20.51	3.79	0.90	0.07	39.69	460.48	2.98	2.20	84.26
**CTU_21_27**	**RR Calypso**	79.11	7.14	27.51	0.34	93.81	2010.66	10.64	4.02	458.60
**CTU_21_28**	**DW686**	11.76	1.86	0.20	0.07	55.01	415.69	2.31	1.42	63.08
**CTU_21_29**	**DJI Spark**	173.46	12.90	129.13	0.35	146.33	2162.07	7.53	8.12	635.16
**CTU_21_30**	**DJI Air**	271.41	23.48	264.84	0.51	192.06	2969.94	10.07	12.43	870.05
**CTU_21_31**	**DJI Mavic Pro 2**	235.40	31.00	356.17	0.76	133.65	4364.75	21.37	5.29	906.36
**CTU_21_32**	**DJI Mavic Pro 2**	302.47	35.87	559.83	0.84	182.41	4820.52	23.58	2.79	1016.99
**CTU_21_33**	**DJI Mavic Pro 2**	279.04	44.64	659.78	0.88	205.66	5061.46	15.64	22.92	1088.40
**CTU_21_34**	**DJI Mavic Pro**	246.94	22.67	263.48	0.66	54.94	3819.87	13.52	22.38	793.32
**CTU_21_35**	**DJI Mavic Pro**	168.50	30.62	185.93	0.61	156.56	3605.77	14.33	3.69	633.97
**CTU_21_36**	**DJI Mavic Pro 2**	247.32	31.99	449.83	0.73	37.26	4235.79	16.49	6.71	918.50
**CTU_21_37**	**DJI Mavic Pro 2**	241.95	36.82	393.44	0.73	41.97	4248.10	18.23	8.82	864.15
**CTU_21_38**	**DJI Phantom 1**	103.98	20.79	45.81	0.58	90.43	3477.84	17.75	2.13	623.90
**CTU_21_39**	**DJI Phantom 3S**	58.90	26.94	43.26	0.55	43.20	3228.16	14.75	3.72	529.45
**CTU_21_40**	**DJI Phantom 3S**	121.62	30.90	107.70	0.70	38.67	4255.19	16.53	9.57	690.11
**CTU_21_41**	**DJI Phantom 2V+**	119.54	21.84	119.50	0.06	48.50	4696.23	18.00	17.22	690.19
**CTU_21_42**	**DJI Phantom 2V+**	130.05	21.00	116.45	0.68	48.22	4221.70	18.51	12.88	656.51
**CTU_21_43**	**DJI Phantom 4 Pro**	55.48	18.92	23.88	0.39	46.36	2041.39	25.90	2.75	460.89
**CTU_21_44**	**DJI Phantom 4 Pro**	70.70	24.18	56.79	0.57	52.20	3313.70	22.62	3.49	632.43
**CTU_21_45**	**DJI Phantom 4 Pro**	89.24	37.46	106.10	0.71	57.87	4361.78	21.26	10.03	784.99
**CTU_21_46**	**DJI Phantom 4 Pro**	73.31	48.86	136.58	0.73	226.51	4533.61	25.45	8.86	790.90
**CTU_21_47**	**DJI Phantom 4 Pro**	212.02	54.83	577.89	0.95	350.83	5917.02	67.73	15.50	1101.42
**CTU_21_48**	**DJI Mavic Pro 2**	69.50	19.66	52.42	0.38	440.62	1052.29	68.47	17.83	755.07
**CTU_21_49**	**DJI Mavic Pro 2**	191.37	29.48	245.17	0.49	335.12	2495.44	37.88	5.53	510.47

**Table 3:** Crash tests results – Estimated severity of injury or probability of fatality.

Internal ID	UAS model	Pof Head (KE-Feinstein)	PoF Head (KE-Janser)	Skull fracture AIS 2 (Peak head acceleration Mertz)	Skull fracture AIS2 (HIC15 Mertz)	Skull fracture AIS2 (HIC36 NHTSA)	Head AIS 3 (HIC15 Prasad)	Head AIS 3 (HIC15 NHTSA)	Brain Injury AIS4 (HIC15 Prasad)	Brain Injury AIS4 (HIC15 Mertz)	AIS 3 injury (Nij)
**Unit**	**-**	**%**	**%**	**%**	**%**	**%**	**%**	**%**	**%**	**%**	**%**
**Limit value**	**-**	**100**	**100**	**100**	**100**	**100**	**100**	**100**	**100**	**100**	**100**
**CTU_21_01**	**Tiny Whoop**	0.00	0.00	0.00	0.11	0.00	0.00	0.00	0.00	0.04	3.97
**CTU_21_02**	**Tiny Whoop**	0.00	0.00	0.00	0.11	0.00	0.00	0.00	0.00	0.04	4.04
**CTU_21_03**	**Tiny Whoop**	0.00	0.00	0.00	0.11	0.00	0.00	0.00	0.00	0.04	3.95
**CTU_21_04**	**Tiny Whoop**	0.00	0.00	0.00	0.11	0.00	0.00	0.00	0.00	0.04	3.84
**CTU_21_05**	**DJI Tello**	0.00	0.00	0.00	0.11	0.00	0.00	0.00	0.00	0.04	4.66
**CTU_21_06**	**DJI Tello**	0.00	0.00	0.00	0.11	0.00	0.00	0.00	0.00	0.05	4.86
**CTU_21_07**	**DJI Tello**	0.00	0.00	0.00	0.11	0.00	0.00	0.00	0.00	0.04	3.97
**CTU_21_08**	**DJI Tello**	0.00	0.00	0.01	0.12	0.00	0.00	0.00	0.00	0.05	5.15
**CTU_21_09**	**DJI Tello**	0.00	0.00	0.00	0.11	0.00	0.00	0.00	0.00	0.05	4.98
**CTU_21_10**	**Eachine E58**	0.00	0.00	0.00	0.11	0.00	0.00	0.00	0.00	0.04	4.23
**CTU_21_11**	**Eachine E59**	0.00	0.00	0.00	0.11	0.00	0.00	0.00	0.00	0.04	4.39
**CTU_21_12**	**Eachine E60**	0.00	0.00	0.00	0.11	0.00	0.00	0.00	0.00	0.04	4.66
**CTU_21_13**	**Syma X5C**	0.00	0.00	0.00	0.11	0.00	0.00	0.00	0.00	0.04	4.28
**CTU_21_14**	**Syma X5C**	0.00	0.00	0.00	0.11	0.00	0.00	0.00	0.00	0.04	4.37
**CTU_21_15**	**Syma X5C**	0.00	0.00	0.00	0.11	0.00	0.00	0.00	0.00	0.04	4.68
**CTU_21_16**	**Syma X5C**	0.00	0.00	0.00	0.11	0.00	0.00	0.00	0.00	0.04	4.63
**CTU_21_17**	**Syma X5C**	0.00	0.00	0.00	0.11	0.00	0.00	0.00	0.00	0.04	4.69
**CTU_21_18**	**Eachine E520s**	0.00	0.00	0.00	0.11	0.00	0.00	0.00	0.00	0.04	4.98
**CTU_21_19**	**Eachine E520s**	4.89	0.48	0.00	0.11	0.00	0.00	0.00	0.00	0.05	5.63
**CTU_21_20**	**Eachine E520s**	32.48	7.17	0.00	0.12	0.00	0.00	0.00	0.00	0.05	5.98
**CTU_21_21**	**Meffie Nano**	0.00	0.00	0.00	0.11	0.00	0.00	0.00	0.00	0.05	4.78
**CTU_21_22**	**Meffie Nano**	0.69	0.04	0.00	0.12	0.00	0.00	0.00	0.00	0.05	5.33
**CTU_21_23**	**RR Calypso**	21.60	3.81	0.05	0.14	0.01	0.21	0.00	0.05	0.06	7.40
**CTU_21_24**	**Cinewhoop**	20.95	3.63	0.07	0.16	0.04	0.49	0.00	0.11	0.07	8.62
**CTU_21_25**	**Cinewhoop**	83.72	45.38	2.77	0.32	1.53	3.50	0.09	0.76	0.16	9.87
**CTU_21_26**	**FOYU FO-F708**	0.00	0.00	0.00	0.11	0.00	0.00	0.00	0.00	0.04	4.39
**CTU_21_27**	**RR Calypso**	11.97	1.62	0.01	0.13	0.00	0.02	0.00	0.01	0.05	7.16
**CTU_21_28**	**DW686**	0.00	0.00	0.00	0.11	0.00	0.00	0.00	0.00	0.04	4.33
**CTU_21_29**	**DJI Spark**	7.09	0.79	3.25	0.25	0.65	2.21	0.02	0.48	0.12	7.33
**CTU_21_30**	**DJI Air**	46.96	13.38	57.77	0.57	5.10	7.50	0.57	1.63	0.33	9.79
**CTU_21_31**	**DJI Mavic Pro 2**	99.29	89.66	28.97	0.95	9.94	13.10	1.65	2.91	0.61	15.12
**CTU_21_32**	**DJI Mavic Pro 2**	99.94	97.75	80.04	2.70	22.63	33.91	6.43	8.74	2.10	17.13
**CTU_21_33**	**DJI Mavic Pro 2**	99.92	97.25	63.87	4.26	28.87	47.53	9.72	14.10	3.59	18.24
**CTU_21_34**	**DJI Mavic Pro**	96.64	75.09	37.69	0.56	5.04	7.43	0.56	1.61	0.32	12.66
**CTU_21_35**	**DJI Mavic Pro**	96.15	73.24	2.57	0.35	2.00	4.09	0.13	0.88	0.19	11.71
**CTU_21_36**	**DJI Mavic Pro 2**	99.41	90.74	37.99	1.57	15.64	21.20	3.47	4.94	1.10	14.19
**CTU_21_37**	**DJI Mavic Pro 2**	99.34	90.04	33.81	1.17	12.15	16.02	2.29	3.61	0.78	14.34
**CTU_21_38**	**DJI Phantom 1**	99.07	87.85	0.05	0.14	0.01	0.20	0.00	0.04	0.06	11.12
**CTU_21_39**	**DJI Phantom 3S**	98.69	85.20	0.00	0.14	0.01	0.17	0.00	0.04	0.06	10.41
**CTU_21_40**	**DJI Phantom 3S**	99.97	98.60	0.17	0.22	0.35	1.60	0.01	0.35	0.10	13.56
**CTU_21_41**	**DJI Phantom 2V+**	99.99	99.23	0.15	0.23	0.50	1.93	0.02	0.42	0.11	4.31
**CTU_21_42**	**DJI Phantom 2V+**	100.00	99.87	0.30	0.23	0.46	1.85	0.01	0.40	0.11	13.10
**CTU_21_43**	**DJI Phantom 4 Pro**	99.44	91.00	0.00	0.12	0.00	0.01	0.00	0.00	0.05	7.82
**CTU_21_44**	**DJI Phantom 4 Pro**	99.99	99.41	0.00	0.16	0.03	0.39	0.00	0.08	0.07	10.96
**CTU_21_45**	**DJI Phantom 4 Pro**	100.00	99.96	0.02	0.21	0.33	1.56	0.01	0.34	0.10	13.79
**CTU_21_46**	**DJI Phantom 4 Pro**	100.00	99.58	0.00	0.26	0.78	2.44	0.03	0.53	0.13	14.23
**CTU_21_47**	**DJI Phantom 4 Pro**	100.00	99.87	14.89	2.94	23.77	36.27	6.98	9.56	2.32	20.51
**CTU_21_48**	**DJI Mavic Pro 2**	99.39	90.48	0.00	0.15	0.02	0.31	0.00	0.07	0.07	7.68
**CTU_21_49**	**DJI Mavic Pro 2**	99.37	90.31	7.06	0.51	4.21	6.54	0.42	1.42	0.28	9.36

Table 7 lists the main parameters of the impact (mass of the impactor, impact speed, achieved KE). Table 8 shows the resulting response from the ATD head and upper neck (peak head acceleration, 3 ms head acceleration, HIC15, Nij and upper neck forces and moments). Depending on the requirements of the input pre-processing of the variables for the individual criteria, the variables were appropriately processed - for example, filtering using the CFC1000 filter for the HIC15 criterion. Other mathematical procedures or adjustments of the data were not applied. Furthermore, the measured values that reach the threshold limit are highlighted in red, while those below are presented as ratios to the threshold values. Table 9 presents the estimated chance of severe injury or predicted Probability of Fatality (PoF).

The probability of a fatality was determined based on the KE probability curves by Feinstein (RCC 321-07) [15,22] and Janser [23]. The limits of skull fracture probability (AIS 2) were estimated based on Peak Head Acceleration [24,25] and HIC15. The AIS 3 head injury probability and ASI 4 brain damage were also determined. The probability of an AIS 3 neck injury was estimated using Nij [25–28].

Overall, the tests correlate well with the tests performed in other studies. The energy-based criteria show significantly higher predicted probabilities of fatality compared to other vulnerability models.

The aim was to validate and assess the repeatability of the tests and compare them to measurements previously performed by the Assure group. To achieve consistent impact velocity, several tests were performed to observe the differences in the ATD response.

Two main types of validation tests were conducted. The first focused on the developed test facility and its procedures, evaluating the ability to reliably assess the parameters of the impact. Therefore, some tests were repeated with identical settings (drop height, impact speed, and resulting KE) to observe the potential differences.

The second type aimed to validate selected tests from the available dataset of the A14 Report (tests UA17A-11/12/13, UA19A-76, and UA19A-45/108). While a certain level of variation was expected due to differences in testing procedures, facilities, and measuring devices, the general results, especially the derived HVM criteria and predicted severity of injury, should correlate.

### 4.1. Validation of the repeatability of developed drop mechanism

For these comparative tests, an identical target impact speed (KE) was used, and the validation was performed through the analysis of the resulting values. In total, three comparative test series were performed. The assessment was conducted by examining variations in relation to the threshold limit values, which are the main factors affecting the predicted injury severity. It is important to note that direct measurements (such as peak head acceleration and the maximum achieved forces and torques in the upper neck) are maximal values that are highly sensitive to even slight deviations in the tests. Thus, larger deviations do not necessarily mean different results. Conversely, the derived HVM criteria, which account for the time effects of the impact or aggregate several measured values, should yield similar values.

**Fig 6:**
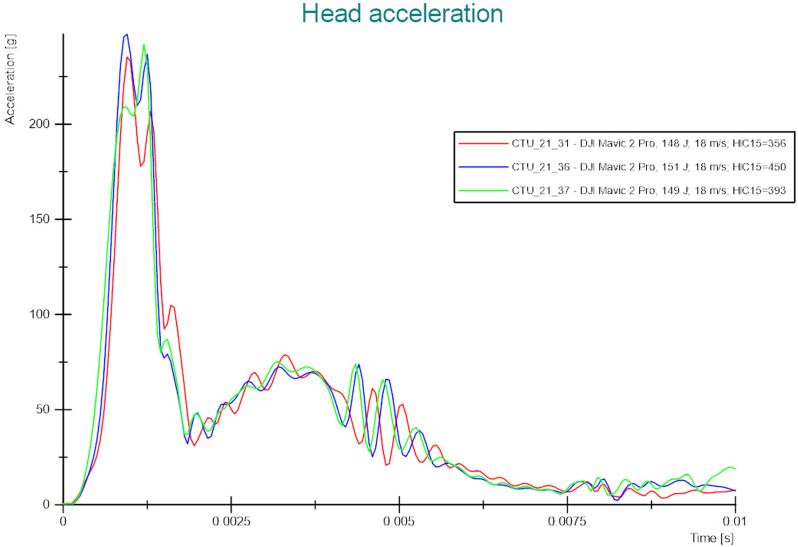
Test results (DJI Mavic 2 Pro) – Head acceleration.

The first comparative test series was performed with a DJI Mavic 2 Pro. The impact configuration involved an impact of the bottom side of the UAS onto the top of the ATD’s head. Three tests were carried out to assess the variability of the measured values on the ATD’s head and upper neck. The target impact speed was set at 18 m/s (KE around 240 J). The results are summarized in Table 4 and can be seen in Fig 6. The achieved impact speeds exhibited very high accuracy (within 1%).

**Table 4:** Test repeatability assessment (Mavic 2 Pro, 18 m/s).

Internal ID	UAS model	UAS mass	Height	Impact velocity	Impact KE	Maximum Resultant Head Acceleration	Head Accel. (3ms)	Head injury criterion (HIC15)	Neck injury criterion (Nij)	Upper neck tension (Fz)	Upper neck compression (-Fz)	Upper neck flexion (-My)	Upper neck extension (My)	Upper neck shear
**Unit**	**- **	**kg**	**m**	**m/s**	**J**	**(g)**	**(g)**	**- **	**-**	**N**	**N**	**Nm**	**Nm**	**N**
**Limit value**	**- **	** -**	**- **	** -**	**80**	**200**	**80**	**700**	**1**	**6806**	**6160**	**310**	**135**	**3100**
CTU_21_31	DJI Mavic 2 Pro	0.917	20.00	17.99	148.32	235.40	31.00	356.17	0.76	133.65	4364.75	21.37	5.29	906.36
CTU_21_37	DJI Mavic 2 Pro	0.913	20.00	18.08	149.28	241.95	36.82	393.44	0.73	41.97	4248.10	18.23	8.82	864.15
CTU_21_36	DJI Mavic 2 Pro	0.920	20.00	18.13	151.12	247.32	31.99	449.83	0.73	37.26	4235.79	16.49	6.71	918.50
**Max Δ to limit value**					**3.49%**	**5.96%**	**7.28%**	**13.38%**	**3.75%**	**1.42%**	**2.09%**	**1.57%**	**2.62%**	**1.75%**
**Max Δ between tests**		**0.76%**	** **	**0.77%**	**1.85%**	**4.82%**	**15.82%**	**20.82%**	**4.92%**	**72.12%**	**2.95%**	**22.82%**	**40.02%**	**5.92%**

Furthermore, the tests showed similar resulting values in relation to the threshold values. The absolute variation is more significant for lower measured values (upper neck tension, extension, and flexion). On the other hand, if the response measured on ATD was higher, the variations were lower. Additionally, the trends in the ATD’s response were consistent across all three tests.

A second comparative test series was performed using a lightweight hobby drone (Syma X5C), which has a high ratio of mass to effective area. This characteristic can impact the accuracy of the performed tests. Due to increased drag and a low terminal velocity, this can lead to potential issues when tested at higher impact speeds. The estimated terminal velocity for this UAS ranged between 7.4 and 9.2 m/s. Therefore, the validation tests were performed with a target impact speed of 18 m/s, double the anticipated speed, to assess the behavior of the test setup and its effects on the measurements. The results show similar trends to those observed in the first comparative test series (Table 5).

**Table 5:** Test repeatability assessment (Syma X5C, 18 m/s).

Internal ID	UAS model	UAS mass	Height	Impact velocity	Impact KE	Maximum Resultant Head Acceleration	Head Accel. (3ms)	Head injury criterion (HIC15)	Neck injury criterion (Nij)	Upper neck tension (Fz)	Upper neck compression (-Fz)	Upper neck flexion (-My)	Upper neck extension (My)	Upper neck shear
**Unit**	**–**	**kg**	**m**	**m/s**	**J**	**(g)**	**(g)**	**–**	**–**	**N**	**N**	**Nm**	**Nm**	**N**
**Limit value**	**–**	**–**	**–**	**–**	**80**	**200**	**80**	**700**	**1**	**6806**	**6160**	**310**	**135**	**3100**
CTU_21_15	Syma X5C	0.099	25.00	18.25	16.48	20.60	4.62	1.29	0.11	85.53	654.34	3.52	3.93	96.47
CTU_21_16	Syma X5C	0.099	25.00	18.35	16.67	17.95	4.51	1.35	0.10	49.19	592.32	3.79	4.54	96.96
CTU_21_17	Syma X5C	0.099	25.00	18.48	16.90	23.69	5.67	1.17	0.11	88.00	621.86	3.68	4.47	89.56
**Max Δ to limit value**					**0.52%**	**2.87%**	**1.45%**	**0.03%**	**0.68%**	**0.57%**	**1.01%**	**0.09%**	**0.46%**	**0.24%**
**Max Δ between tests**		**0.00%**		**1.23%**	**2.45%**	**24.21%**	**20.43%**	**13.02%**	**6.21%**	**44.11%**	**9.48%**	**7.16%**	**13.61%**	**7.63%**

The third comparative series consisted of two pairs of tests for different UASs. The first was for the smaller DJI Tello drone and the second for the DJI Phantom 4 Pro. The aim was to assess the capability of the developed test facility to be used for various sizes and weights of test articles. The results are presented in Table 6. Although nearly identical values were observed for the DJI Tello, the Phantom 4 Pro test exhibited greater variation. This variation can be attributed to the minimal effects of the lightweight drone on the ATD. However, the measured values for the second impact of the Phantom 4 Pro were higher, even when considering the differences in impact speed and KE difference is considered. This deviation was not observed in other comparative test series. One possible explanation is that the variation may be partly attributed to the different positions of the camera in the final moments before impact [29]. While it is acknowledged that slight deviations in the center of gravity (CG) alignment during impacts can affect energy transmission, the camera in the DJI Phantom 4 impact may influence the resulting load on the ATD. Slight offsets caused by the orientation of the rigid components (camera and gimbal) can lead to different loading factors.

**Table 6:** Test repeatability assessment (DJI Tello, 18 m/s and Phantom 4 Pro, 18 m/s).

Internal ID	UAS model		UAS mass	Height	Impact velocity	Impact KE	Maximum Resultant Head Acceleration	Head Accel. (3ms)		Head injury criterion (HIC15)	Neck injury criterion (Nij)	Upper neck tension (Fz)	Upper neck compression (-Fz)		Upper neck flexion (-My)	Upper neck extension (My)	Upper neck shear
**Unit**	**–**		**kg**	**m**	**m/s**	**J**	**(g)**	**(g)**		**–**	**–**	**N**	**N**		**Nm**	**Nm**	**N**
**Limit value**	**–**		**–**	**–**	**–**	**80**	**200**	**80**		**700**	**1**	**6806**	**6160**		**310**	**135**	**3100**
CTU_21_08	DJI Tello		0.086	20.00	17.81	13.65	84.19	3.33		13.58	0.16	116.34	762.99		4.12	6.87	209.28
CTU_21_09	DJI Tello		0.086	21.00	18.47	14.66	74.04	2.53		11.90	0.14	100.17	749.83		3.94	5.16	199.05
**Max Δ to limit value**						**1.27%**	**5.07%**	**1.00%**		**0.24%**	**1.73%**	**0.24%**	**0.21%**		**0.06%**	**1.26%**	**0.33%**
**Max Δ between tests**			**0.00%**		**3.66%**	**7.46%**	**12.05%**	**24.06%**		**12.31%**	**10.91%**	**13.90%**	**1.72%**		**4.50%**	**24.85%**	**4.89%**
CTU_21_44		DJI Phantom 4 Pro	1.386	20.00	17.65	215.95	70.70	24.18		56.79	0.57	52.20		3313.70	22.62	3.49	632.43
CTU_21_46		DJI Phantom 4 Pro	1.382	20.00	18.00	223.88	73.31		48.86	136.58	0.73	226.51		4533.61	25.45	8.86	790.90
**Δ to limit value**						**9.91%**	**1.31%**		**30.84%**	**11.40%**	**15.20%**	**2.56%**		**19.80%**	**0.91%**	**3.98%**	**5.11%**
**Δ between tests**			**0.29%**		**1.97%**	**3.67%**	**3.70%**		**102.02%**	**140.51%**	**26.44%**	**333.94%**		**36.81%**	**12.51%**	**154.05%**	**25.06%**

In conclusion, the developed test facility has proven capable of reliably performing measurements, not only in terms of impact speed but also regarding the desired impact configuration. Furthermore, the drop sled can be used to assess a wide range of multi-copter UASs with varying mass and impact speeds. This capability is crucial for assessing various UAS types based on the conducted tests. Based on the last comparative series, if the camera and other movable but rigid objects collide with the ATD, it may introduce a certain level of variation in the test results.

### 4.2. Validation against the selected A14 datasets

The validation was performed for three tests in total. The assessment was based on a comparison of the achieved dynamic response of the ATD head and upper neck, as well as derived automotive HVM criteria (HIC15, Nij). Two types of percentual deviations were assessed. The first type was similar to previous validations of the drop mechanism. The second was the mutual variations among the absolute measured values.

The first selected validation test was a vertical impact of DJI Phantom 3 Standard (target impact speed of 15 m/s, KE of 136 J) impacting the top of the ATD head with its underside (Table 7). The three Assure tests were combined to create an average, which was subsequently compared. Variations for the averaged values were also conducted to show how the measured values can differ even with the same setup and test configuration. Overall, there is a strong correlation between the tests, where none of the values reach the threshold limits. Furthermore, similar ranges of measured values can be identified in relation to the threshold limits. However, the authors of the tests indicate a slightly increased risk to the neck and lower risk to the head. This may be attributed to deviations in the position of the ATD, which are not extensively detailed in the A14 Report.

**Table 7:** Tests 39 - Validation test against the UA17A-11/12/13 tests.

Internal ID	UAS model	UAS mass	Height	Impact velocity	Impact KE	Maximum Resultant Head Acceleration	Head Accel. (3ms)	Head injury criterion (HIC15)	Neck injury criterion (Nij)	Upper neck tension (Fz)	Upper neck compression (-Fz)	Upper neck flexion (-My)	Upper neck extension (My)	Upper neck shear
**Unit**	**–**	**kg**	**m**	**m/s**	**J**	**(g)**	**(g)**	**–**	**–**	**N**	**N**	**Nm**	**Nm**	**N**
**Limit value**	**–**	**–**	**–**	**–**	**80**	**200**	**80**	**700**	**1**	**6806**	**6160**	**310**	**135**	**3100**
**CTU_21_39**	**DJI Phantom 3 Standard**	1.22	13.00	15.07	138.98	58.90	26.94	43	0.55	43	3228	15	4	529
UA17A-11	Phantom 3 Standard	1.21	15.24	15.11	138.29	82.38	–	60	0.65	195	3675	9	21	214
UA17A-12	Phantom 3 Standard	1.213	0	14.99	136.27	71.50	–	48	0.62	188	3455	10	21	208
UA17A-13	Phantom 3 Standard	1.211	0	14.98	135.85	119.13	–	42	0.63	115	3352	10	23	279
**Mean of Assure tests**		**1.212**		**15.03**	**136.80**	**91.00**	**–**	**50**	**0.63**	**166**	**3494**	**10**	**22**	**234**
Δ **Assure (related to limit value)**					**3.05%**	**23.82%**		**2.47%**	**3.00%**	**1.19%**	**5.26%**	**0.30%**	**1.27%**	**2.28%**
Δ **Assure tests (absolute)**		**0.19%**		**0.89%**	**1.77%**	**39.98%**		**29.06%**	**4.62%**	**41.28%**	**8.81%**	**8.98%**	**7.54%**	**25.35%**
Δ **CTU to mean (related to limit value)**					**2.72%**	**‒16.05%**	**–**	**‒0.95%**	**‒8.77%**	**‒1.80%**	**‒4.32%**	**1.51%**	**‒13.29%**	**9.54%**
Δ **CTU to mean (absolute)**		**0.99%**		**0.29%**	**1.57%**	**‒54.51%**	**–**	**‒15.43%**	**‒16.08%**	**‒284.28%**	**‒8.24%**	**31.78%**	**‒481.84%**	**55.84%**

As a second validation test, a vertical impact of the DJI Mavic Pro (target impact speed of 18 m/s, KE of 115 J) impacting the top of the head of the ATD’s head with its top side (Table 8) was selected. For this test, only one corresponding test is available in the Assure database. While the target impact speed for the authors’ test was met, the slightly heavier test article resulted in a higher impact KE (7%). Similarly to the first validation test, none of the threshold limits were exceeded, and the overall correspondence is good. However, the authors’ test also predicted slightly more severe risks to the neck and lower risks to the head.

**Table 8:** Tests 34 - Validation test against the UA19A-76 test.

internal ID	UAS model	UAS mass	Height	Impact velocity	Impact KE	Maximum Resultant Head Acceleration	Head Accel. (3ms)	Head injury criterion (HIC15)	Neck injury criterion (Nij)	Upper neck tension (Fz)	Upper neck compression (-Fz)	Upper neck flexion (-My)	Upper neck extension (My)	Upper neck shear
**Unit**	**–**	**kg**	**m**	**m/s**	**J**	**(g)**	**(g)**	**–**	**–**	**N**	**N**	**Nm**	**Nm**	**N**
**Limit value**	**–**	**–**	**–**	**–**	**80**	**200**	**80**	**700**	**1**	**6806**	**6160**	**310**	**135**	**3100**
**CTU_21_34**	**DJI Mavic Pro**	0.75	20.00	18.22	124.52	246.94	246.94	263.48	0.66	54.94	3819.87	13.52	22.38	793.32
**UA19A-76**	**Mavic Pro**	0.70		18.19	115.68	241.54	241.54	298.15	0.59	203.19	3569.88	16.26	4.99	534.32
**Max Δ to limit value**					**11.05%**	**2.70%**	**6.75%**	**4.95%**	**6.78%**	**2.18%**	**4.06%**	**0.88%**	**12.88%**	**8.35%**
**Max Δ between tests**		**-7.23%**		**0.19%**	**7.10%**	**2.19%**	**2.19%**	**11.63%**	**10.31%**	**72.96%**	**6.54%**	**16.84%**	**77.71%**	**32.65%**

Lastly, the third validation was performed. While the main focus of the testing was on vertical impacts to the top of the ATD’s head, an effort was made to assess the setup’s capability for angled impacts with the ATD inclined. Therefore, the final validation test involved an angled impact of a DJI Phantom 3 (target impact speed of 20 m/s, KE of 250 J) impacting the front of the ATD’s head at a 58° angle, with the UAS aligned between its arms (Table 9). For this test, two similar tests were available in the A14 Report, but they involved two different UASs (Phantom 3 Standard and Professional). However, these tests, differed in the impact speed and UAS weight. Therefore, it was decided to conduct the test with the last remaining Phantom 4 Pro available and to adjust the impact configuration to the average of those two tests. Furthermore, in the case of the Assure tests, the UAS was launched via pneumatically actuated launcher to the ATD in a normal sitting position. In the authors’ tests, the UAS was dropped vertically onto an inclined ATD. However, the comparison of the tests revealed similar trends and criterion values. Both tests showed HIC15 and Nij values near the threshold limits for head and neck injuries, with the primary effect being upper neck compression.

**Table 9:** Tests 47 - Validation test against the UA19A-45/108 tests.

Internal ID	UAS model	UAS mass	Height	Impact velocity	Impact KE	Maximum Resultant Head Acceleration	Head Accel. (3ms)	Head injury criterion (HIC15)	Neck injury criterion (Nij)	Upper neck tension (Fz)	Upper neck compression (-Fz)	Upper neck flexion (-My)	Upper neck extension (My)	Upper neck shear
**Unit**	**–**	**kg**	**m**	**m/s**	**J**	**(g)**	**(g)**	**–**	**–**	**N**	**N**	**Nm**	**Nm**	**N**
**Limit value**	**–**	**–**	**–**	**–**	**80**	**200**	**80**	**700**	**1**	**6806**	**6160**	**310**	**135**	**3100**
**CTU_21_47**	**DJI Phantom 4 Pro**	1.381	20.00	19.04	**250.36**	212.02	54.83	577.89	0.95	350.83	5917.02	67.73	15.50	1101.42
**UA19A-45**	**Phantom 3 Professional**	1.218		19.64	234.85	191.06	58.80	446	0.77	314	4260	49	19	1246
**UA19A-108**	**Phantom 3 Standard**	1.148		21.89	275.04	363.70	77.28	1064	0.89	221	5280	22	16	1420
**Mean of Assure tests**		**1.183**		**20.19**	**254.94**	**254.94**	**64**	**696**	**0.87**	**295**	**5152**	**46**	**17**	**1256**
**Δ Assure (related to limit value)**					**50.24%**	**86.32%**		**88.20%**	**12.00%**	**1.36%**	**16.56%**	**8.61%**	**2.34%**	**5.59%**
**Δ Assure tests (absolute)**		**5.77%**		**10.30%**	**14.61%**	**47.47%**		**58.03%**	**13.48%**	**29.46%**	**19.32%**	**54.32%**	**16.92%**	**12.21%**
**Δ CTU to mean (related to limit value)**					**‒5.72%**	**‒21.79%**		**‒16.88%**	**8.06%**	**0.82%**	**12.42%**	**6.87%**	**‒0.79%**	**‒4.98%**
**Δ CTU to mean (absolute)**		**9.57%**		**‒6.04%**	**‒1.83%**	**‒20.55%**		**‒20.45%**	**8.48%**	**15.85%**	**12.93%**	**31.43%**	**‒6.85%**	**‒14.01%**

Overall, the validation showed a high level of correspondence between the tests. The observed values, which were similar or very close, indicate that the drop testing apparatus developed by DFET was able to reproduce results comparable to those achieved by Assure’s facilities. Deviations of the ATD posture were observed, and the HIC15 appears to be a good predictor with a higher degree of robustness. The DFET testing facility tended to show slightly lower values for head acceleration and higher values for neck loading, which may be attributed to the unknown exact position of the ATD during the tests. Although the exact position of the ATD is not covered in the current standards that set the parameters for UAS crash testing, it affects the results, and should therefore be defined in greater detail in the future. Lastly, the results of the angled impact validation indicate that it is possible to obtain similar outcomes by positioning the ATD at an angled launcher, as suggested in Appendix C of AC 922-001.

### 4.3. Peak Head Acceleration and HIC15 (DJI Phantom)

**Fig 7:**
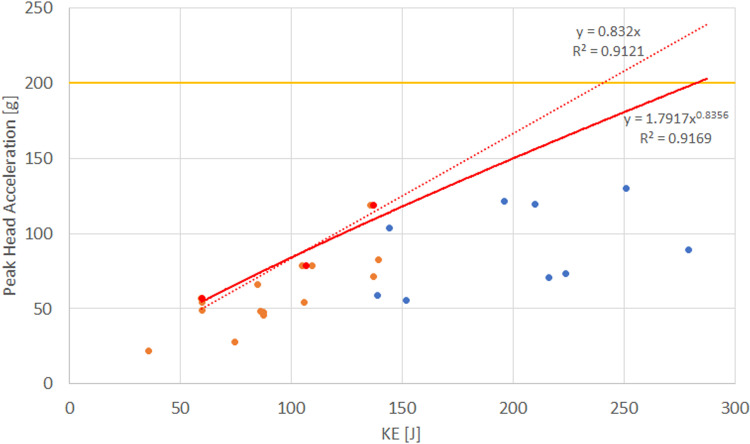
Energy transfer slope (linear trend shown as a dotted line) based on ASTM Method C for DJI Phantom impacts. Maximum values of peak head acceleration at 10, 13, and 15 m/s are marked (red dots); CTU test data (blue dots); Assure A4 and A14 test data (orange dots); 200 g limit (solid orange line); power trend fitting (solid red line).

**Fig 8:**
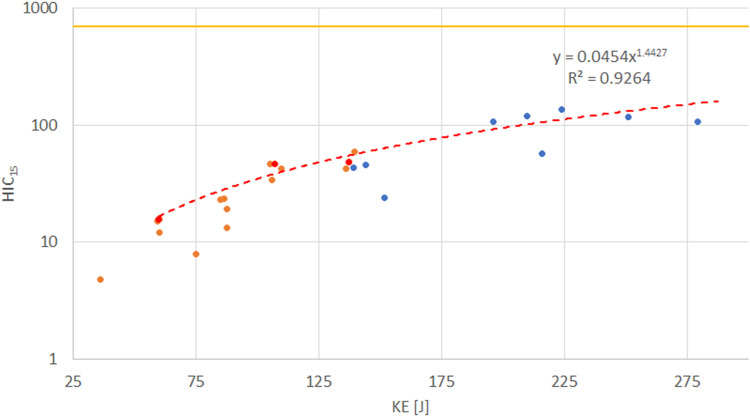
Predicted HIC_15_ values (dashed line) for DJI Phantom impacts. Maximum values of peak head acceleration at 10, 13, and 15 m/s are marked (red dots); CTU test data (blue dots); Assure test data (orange dots); 700 HIC15 limit (solid orange line).

The advantage of utilizing a broader dataset of complementary data is evident when examining the relationship between peak head acceleration and impact KE. By combining test data for DJI Phantom 3 and 4 drones—similar in design—obtained from available sources (A4 and A14 tests) with results from the present study, limitations in the linear relationship between peak head acceleration and KE become apparent. Figs 7 and 8 illustrate vertical impacts of the DJI Phantom on the ATD head, showing the achieved KE, peak head acceleration, and HIC_15_ values.

The observed results reveal a non-linear trend for peak head acceleration with significant variability compared to HIC15. For example, applying ASTM Method C to the combined dataset at three impact speeds (10, 13, and 15 m/s), with three tests at each speed, yields an energy transfer slope and a maximum safe impact energy (Fig 7). When this slope is extrapolated to a terminal velocity of 20 m/s (KE of 244 J), the predicted peak head acceleration is 203 g using a linear trend and 177 g using a power trend. Both values exceed the actual measured value from a test at 250 J KE (CTU_21_42), which recorded a peak head acceleration of 130 g.

Additionally, the peak head acceleration values for 15 m/s impacts display significant variation (72 g, 104 g, and 119 g). Standard crashworthiness testing practices typically account for such variability by omitting extreme values or using averaged results to mitigate measurement bias. Relying on the maximum value for predictions risks overly restrictive estimates, which may be suitable for generalized safety guidelines but could be inefficient for cost-effective, detailed testing.

When similar principles are applied to HIC_15_ predictions (Fig 8), which can also be derived from peak head acceleration, the outcomes are more consistent and accurate even with limited data. HIC15 exhibits an asymptotic trend with increasing KE, suggesting that the energy transmitted to the ATD decreases progressively as KE rises. This phenomenon likely arises from the drone’s structural fragility; as the plastic frame undergoes extensive deformation and fracturing, its ability to transfer energy to the head diminishes.

## 5. Discussion

There is a positive trend in the availability of information that enables us to better understand the issue of UAS collision with human. Computational simulation represents the most suitable solution for rapid validation and safety assessment. This allows for easily modification of tested scenarios, observation of the effects of probable impact configurations, and the ability to repeat or validate various measurements. However, the accuracy and validity of such simulations are inherently dependent on the models used, as well as the available information about the UAS, its characteristics, and their validation against performed crash tests. This limitation restricts their interpretive value to some extent.

An important issue identified is that most conclusions, proposed requirements, and test procedures are primarily derived from a single source of information, the Assure research. Other published research in this field does not provide alternative solutions or sufficiently comprehensive data for practical application. While the contribution of Assure is undeniably valuable, the data obtained are highly informative, and the developed and proposed procedures represent a significant advancement in this area, the test results have never been validated by other researchers or testing facilities. The associated costs, time demands, equipment and necessary knowledge present a significant limitation to the possibility of verification. Furthermore, the ASTM standard states, that the reproducibility of Methods B, C, and D for measuring is not provided due to limited availability of testing apparatus and sufficient test sites, which is expected to improve in the future. This raises potential issues with proposed HVM criteria, suggested threshold values, or the application of automotive criteria and ATDs to fields for which they were not specifically designed.

While the tests performed in this study had limitations due to the restricted impact configurations and the use of an ATD primarily designed for automotive testing, they nonetheless broadened the available data and highlighted several important factors. The ability to reproduce test is highly dependent on the clear definition of impact parameters, especially the position and orientation of the ATD and UAS during the impact and the impact location. As in previous studies, the effects of even slight offsets or differences can lead to a significant variations in the observed response. There is also a significant discrepancy between the biomechanical and kinetic prediction models. The most probable reason is the amount of transmitted kinetic energy, which is influenced by the structural characteristics of the UAS compared to the projectiles used to derive the kinetic energy models. While mass and the corresponding kinetic energy present easily definable threshold, the available datasets and performed tests show limitations of their predictive ability for deformable and frangible structures of UASs. Lastly, the use of peak head acceleration or the energy transfer slope as the primary indicators for predicting injury severity presents several potential issues. Peak head acceleration exhibits greater variation than HIC and the assumed linear relation with KE, as illustrated in the case of the DJI Phantom. While the linear trend may be suitable for describing the relationship between peak head acceleration and KE for a rigid impactor, the proposed methodology may lead to overly restrictive prediction when applied to the more pliable and deformable structures of UASs, especially if the tests are not conducted at the maximum impact speed.

## 6. Conclusion

This study expanded the understanding of unmanned aerial system collision dynamics through a series of controlled drop tests. A newly developed drop mechanism facilitated precise and repeatable testing, enabling the collection of high-fidelity data on impact forces and injury risks associated with various UAS types (masses ranging from 20 g to over 1 kg, with impact speeds of 7–24 m/s). By validating existing methodologies against prior datasets and their extension, this research confirmed discrepancies in predictions based on kinetic energy and biomechanical criteria. Furthermore, the extension of available data identified key limitations in the predictive reliability of the energy transfer slope (linear trend fitting) which leads to a significant over-restrictiveness. Importantly, the findings underscore the value of comprehensive metrics, such as the Head Injury Criterion, which accounts for the temporal profile of impacts and provides a more robust measure of injury potential.

Future research should prioritize expanding testing configurations to include horizontal and angled impacts to capture a broader range of real-world scenarios. Additionally, further investigation into energy transfer mechanisms, particularly the observed discrepancies between KE models and biomechanical criteria predictions, is essential. Developing alternative testing procedures with reduced time and financial demands—such as those focused on quantifying the ratio of transferred energy in relation to UAS design, materials, and structures—could significantly enhance the availability of critical data. Achieving these advancements will require harmonized protocols for testing facilities, refined biomechanical injury models, and collaborative efforts to ensure data compatibility and reproducibility. Such developments are crucial for advancing UAS safety research and informing the creation of more effective regulatory frameworks.
